# A Prospective Study Assessing Tumour Response, Survival, and Palliative Care Outcomes in Patients with HIV-Related Kaposi's Sarcoma at Queen Elizabeth Central Hospital, Blantyre, Malawi

**DOI:** 10.1155/2012/312564

**Published:** 2012-03-11

**Authors:** H. Francis, M. J. Bates, L. Kalilani

**Affiliations:** ^1^Department of Internal Medical Services, Ballarat Health Services, Ballarat, VIC 3350, Australia; ^2^Tiyanjane Clinic, Queen Elizabeth Central Hospital, C/- University of Malawi College of Medicine, Private Bag 360, Chichiri, Blantyre 3, Malawi; ^3^University of Malawi, College of Medicine, Private Bag 360, Chichiri, Blantyre 3, Malawi

## Abstract

*Background*. Human-Immunodeficiency-Virus- (HIV-) related Kaposi's sarcoma (KS) has a high prevalence in Africa; however, there is minimal published data on treatment and outcomes in this population. *Objective and Design*. This was a prospective study of 50 patients, aiming to assess the impact of vincristine therapy on tumour response and survival and to assess palliative care outcomes in patients with HIV-related KS. *Methods*. 50 consecutive patients were recruited during 2008. Vincristine therapy and highly active antiretroviral therapy (HAART) were given. Tumour response, survival, and chemotherapy-related toxicities were documented. Palliative care outcomes were assessed using the African Palliative Care Association (APCA) Palliative Outcome Scale (POS). *Results*. The majority of patients were male, and the median age was 33 years. At baseline assessment, the median CD4 T-cell count was 263, and 50% patients had evidence of peripheral neuropathy. The overall response rate was 64% at 6 weeks, and median progression-free survival was 30 weeks. Treatment was generally well tolerated, with peripheral neuropathy the main dose-limiting toxicity. *Conclusion*. The combination of vincristine and HAART is feasible and effective in a low resource setting, although peripheral neuropathy is a dose-limiting factor. This patient group carries a high mortality and as such adequate access to palliative care is crucial.

## 1. Introduction

Kaposi's sarcoma (KS) is the most common human-immunodeficiency virus- (HIV-) related malignancy, and the most common malignancy in Malawi, explaining at 54% of cancer diagnoses in males, and 27% in females from 1994–1998 [1]. Although widespread access to highly active antiretroviral therapy (HAART) has resulted in the reduction in prevalence of KS in the developed world [2, 3], there is minimal data regarding the impact of increased access to HAART in Sub-Saharan Africa. KS accounted for 5–10% of new registrations for HAART in Malawi in 2005 and was associated with a poorer outcome than for other patients on HAART [4]. Since antiretroviral therapy became available to patients in Malawi from 2004, a fixed dose combination of nevirapine, stavudine, and lamivudine has been utilized as first line therapy for HIV.

Malawi is one of the poorest countries in Sub-Saharan Africa and has an estimated HIV seroprevalence of approximately 12% [5]. Delivery of appropriate health care in these circumstances presents a significant challenge, with a lack of human resource, unpredictable drug stocks, and poor laboratory facilities. Infrastructure and expertise required to administer and support combination chemotherapy are lacking in most facilities. Until recently, HAART was available only to patients in industrialized countries; however, by December 2005, over 800,000 patients had been commenced on HAART in Africa [6]. Guidelines published by the Government of Malawi Ministry of Health advocate the use of single agent vincristine chemotherapy in association with HAART for the management of moderate-advanced KS [7]. This approach is utilized in other similar settings, although there is a paucity of data relating to the objective response rate of this treatment regimen.

Tiyanjane Clinic, at Queen Elizabeth Central Hospital in Blantyre, Malawi, provides an outpatient palliative care service and is a referral point for patients with moderate- advanced cases of KS. Pharmacological therapy is given alongside holistic care and support, where psychological, social, and spiritual issues are discussed and explored. The overall aim of the service is to improve the quality of life of those with advanced disease.

Although palliative care services in Sub-Saharan Africa are growing and improving, assessment of such services (as with palliative care services world-wide) remains challenging and outcomes largely unknown [8, 9]. The African Palliative Care Association (APCA) Palliative Outcome Scale (POS), in the appendix [10], is a simple and brief multidimensional outcome measure intended to assist in measuring palliative outcomes in routine clinical practice. 

## 2. Objectives

This was a prospective study to assess the impact of vincristine therapy on tumour response and survival and to assess the feasibility of utilizing the APCA POS in patients with HIV-related KS. Chemotherapy related toxicities were documented.

## 3. Methods

Fifty patients being treated at the Tiyanjane Clinic were recruited from April 2008 to December 2008. All patients referred to the clinic that satisfied inclusion criteria were offered enrollment in the study and none declined. Informed consent was obtained from all patients at the time of enrollment, with thumbprints taken from those unable to write. Ethics approval was obtained from the College of Medicine Research Ethics Committee, College of Medicine, University of Malawi.

Patients were eligible if they were aged 18–70, HIV seropositive, had a clinical diagnosis of Kaposi's sarcoma, and fulfilled criteria for vincristine according to the Malawi National Guidelines [7], those being as follows:

- presumed/confirmed visceral disease,
- cutaneous disease causing symptoms significant enough to impair function,
- progressive disease despite use of ART.

Patients were excluded if they had significant preexisting myelosuppression (baseline hemoglobin levels <8 g/dL and a platelet count of <50/mm^3^), preexisting grade 3 or 4 peripheral neuropathy, clinical jaundice or known chronic liver disease, pregnancy, or had received chemotherapy or radiotherapy within last 6 weeks. Those considered to have a life expectancy of less than 2 weeks and those with active opportunistic infection (other than patients stable on tuberculosis treatment) were also excluded.

Baseline assessment included detailed history, physical examination, full bloods count, and CD4 count. Further CD4 counts were not routinely performed given logistical and funding constraints. Vincristine was given intravenously at a dose of 2 mg weekly for 6 doses, followed by 2 mg fortnightly for 6 doses, followed by 2 mg monthly until progression or unacceptable toxicity. At each consultation, holistic palliative care was administered focusing on physical, psychosocial, and spiritual issues. The treating team included a UK-trained palliative care physician, an Australian medical oncologist, and local nursing staff trained in palliative care. All patients had WHO stage IV HIV by definition [11].

### 3.1. Outcome Measures

The primary outcome measure was tumour response. Patients were defined as having complete response (CR), partial response (PR), stable disease (SD), or progressive disease (PD) as defined by the AIDS Clinical Trials Group (ACTG) response criteria [12], utilizing serial measurements and photographs of indicator lesions. Secondary endpoints were progression-free survival (PFS), defined as the time from study enrolment to either progression or death from any cause, chemotherapy-related toxicity, and palliative care outcomes as measured using the APCA POS tool. The APCA POS consists of a 10-point questionnaire covering physical, psychological, and spiritual domains. Although this tool is able to be self-administered, those patients unable to read or write had the questions read to them by a study nurse. Patients were given a choice of responding to either the Chichewa or English version of the tool.

Chemotherapy-related toxicities were classified according to the Common Terminology Criteria for Adverse Events (Version 3.0, 2006).

### 3.2. Followup

Patients were reviewed every 3 doses of vincristine, which equated to a review at 3 weeks, 6 weeks, 12 weeks, 18 weeks, and 30 weeks. Tumour response and chemotherapy toxicities were recorded at each review. An APCA POS questionnaire was completed at each review.

### 3.3. Translational

The APCA POS tool, patient information leaflet, and informed consent form were translated from English to Chichewa by clinical staff fluent in both Chichewa and English. The standard methodology of translation, backtranslation, and resolution of the differences between back translation and the original was used.

### 3.4. Data Management and Analysis

Microsoft access was used to record and analyze data. Each study proforma was entered as a separate table linked in the database by the unique identifier assigned to each study subject. Personal identifiers were maintained separately in one main file in a password-protected computer file and used only as necessary to match this information with the other databases. Only the investigators of the study had access to this information, which is securely stored and will be destroyed after seven years. Descriptive statistics are used to describe most results. Survival curves are presented and both intention-to-treat (ITT) and per-protocol analyses of median PFS are presented. The Mann-Whitney *U* test was used to explore associations between baseline CD4 counts and outcomes.

## 4. Results

Baseline demographic data are recorded in Table 1. Most patients were male and aged between 28 and 41. Baseline median Karnofsky performance status (KPS) [13] was 70 (range 50–80), indicating that most patients were self-caring however somewhat limited in their ability to perform active work. 50% patients had a preexisting peripheral neuropathy, which was predominantly sensory and grade 1. Three patients had grade 2 neuropathy at baseline.

All patients had clinical evidence of cutaneous KS. 40 patients (80%) had oral cavity involvement, and 31 (62%) had clinical evidence of nodal involvement. 9 patients (18%) had suspected visceral involvement, with 3 of these confirmed with either bronchoscopy or gastroscopy. The majority (37, 74%) of patients had been commenced on HAART prior to commencement of the study, for a median of 6.8 months. A further 8 patients commenced HAART after study enrollment. All patients receiving HAART received the standard regimen available in Malawi consisting of nevirapine, stavudine, and lamivudine.

45 patients were evaluable at 3 weeks, 40 at 6 weeks, 36 at 12 weeks, 33 at 18 weeks, and 25 at 6 months. 16 (32%) patients died during the study period and 9 (18%) defaulted despite intensive efforts at followup (including home visits and telephone calls). At 6 weeks, PR was seen in 32 (64%) patients, and stable disease in 14%, for a clinical benefit rate of 78%. At 12 weeks, ongoing PR was seen in 26 (52%) patients and a further 4 had stable disease. The vast majority of patients were taking oral HAART in addition to receiving vincristine, and so the above response rates reflect combination treatment with HAART and vincristine rather than vincristine alone.

**Figure 1:**
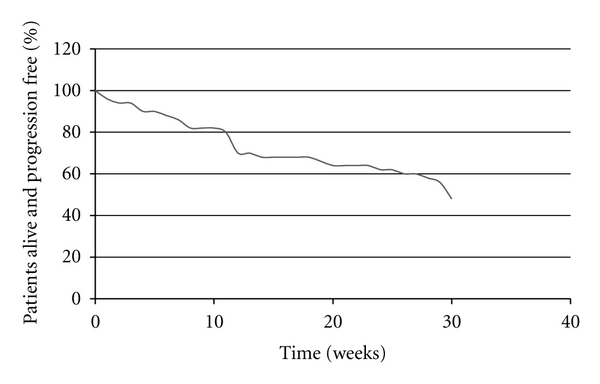
Progression-free survival (intention-to-treat analysis).

**Figure 2:**
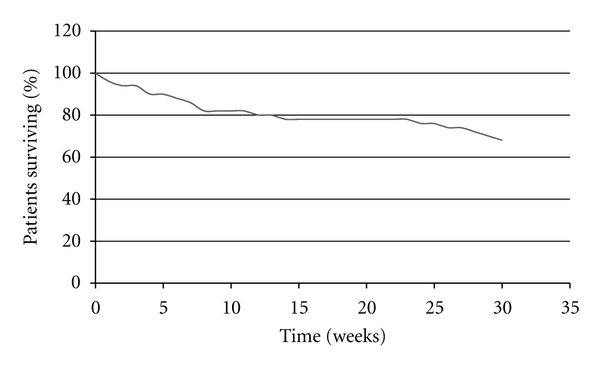
Overall survival.

16 patients died during the study period. PFS data are presented in Figure 1. Median PFS in the ITT population was 30 weeks (i.e., just reached at the end of the study followup period). Overall survival (OS) data are presented in Figure 2. Median OS had not yet been reached at the end of the followup period.

There was no association between baseline CD4 count and disease progression (median CD4 333 versus 256 in progressors and nonprogressors, respectively, *P* = 0.13). However, there was a trend towards a lower CD4 count in those who died during study followup (149 versus 290 in those dead and alive, respectively, *P* = 0.09).

Peripheral neuropathy was documented in 25 of 50 patients at baseline, and 20 of 33 evaluable patients at 18 weeks. Vincristine was ceased in 8 patients due to worsening neuropathy (after a median of 6.5 doses). No significant myelosuppression occurred.

**Figure 3:**
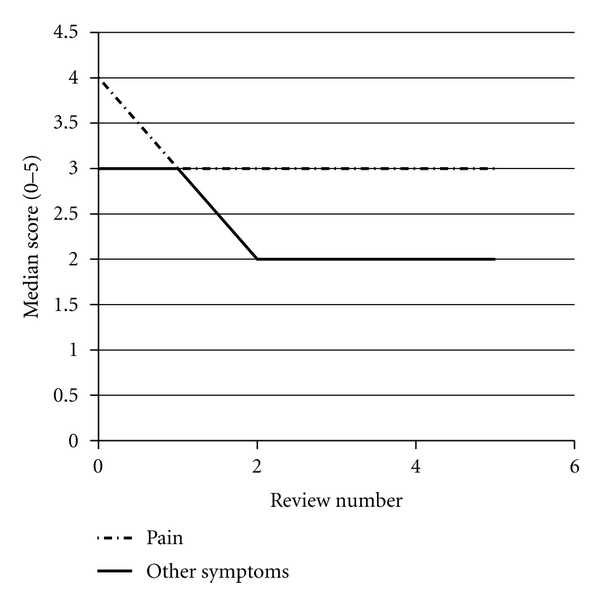
Changes in median APCA POS scores for pain and symptom control over time using the APCA POS. This graph depicts the changes in median scores for questions 1 and 2 from baseline to review number 5.

**Figure 4:**
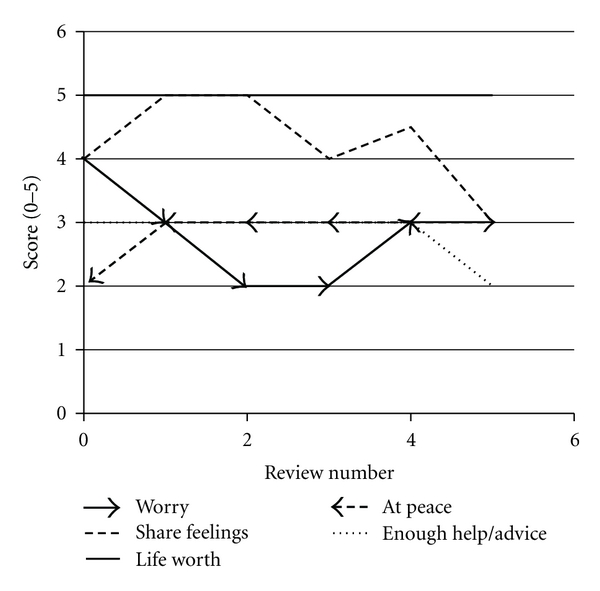
Changes in median APCA POS score for psychosocial domains. This graph depicts the changes in median scores for questions 3–7, from baseline to review number 5.

The baseline APCA POS assessment revealed moderate levels of need in most domains (pain, other symptoms, level of worry). Median pain scores and other symptom scores showed a trend towards improvement over time (Figure 3). Most responded positively to a question of life being worthwhile, and this did not alter during the course of the study (Figure 4). Median scores for a question of feeling at peace were low, as were scores regarding patients feeling they had been given sufficient advice for the future. These domains did not improve significantly during the study.

## 5. Discussion

This study is one of the few looking at patient outcomes in HIV-associated KS, in the era of widespread antiretroviral therapy in the developing world. We found that the combination of HAART and vincristine therapy is associated with an impressive response rate, although there were no reports of complete response in our study. Liposomal doxorubicin is considered the standard of care in the developed world on the basis of two large randomized clinical trials [14, 15]; however, it is not only expensive, but can be associated with myelosuppression and gastrointestinal side effects which would be difficult to manage currently in our setting. Northfelt et al. reported 1 complete response and 60 partial responses out of 133 patients who received liposomal doxorubicin, for an overall response rate of 45.9% in their study [14]. Median time to response was 39 days. The highly favourable response rate in our series is likely explained by a combination effect of vincristine, along with immune reconstitution associated with HAART.

The difficulties in distinguishing the antitumour effects of HAART from the effects of chemotherapy have been previously described [16]. The FDA has previously suggested that patients should receive an extended period of HAART prior to being considered for chemotherapy. The Malawian guidelines, for the use of vincristine described above, are consistent with this suggestion. In our study, 74% patients were receiving HAART for a median of 6.8 months prior to commencing vincristine, which was consistent with these guidelines.

There are few studies looking at the use of chemotherapy for HIV-associated KS in Sub-Saharan Africa. Olweny et al. [17] report on a randomized study of 495 patients in Zimbabwe comparing two chemotherapy regimens, radiotherapy and best supportive care. No complete responses were seen, however single agent etoposide was associated with improved quality of life, and a partial response rate of 31%. Combination chemotherapy (actinomycin-D, bleomycin, vincristine) was associated with a response rate of 49%, however with considerable toxicity (alopecia, mucositis, nausea/vomiting). No patient on this study received HAART. Bihl et al. report on a small South African study of 33 patients, randomized to HAART alone or HAART in combination with combination chemotherapy (doxorubicin, bleomycin, vincristine) [18]. Both arms produced a profound decline in KSHV viraemia and improved CD4 counts. 16 patients received HAART and chemotherapy, with 7 (44%) achieving a complete response and 7 (44%) achieving partial response when measured at 11 months, however the authors acknowledge considerable toxicity of this regimen.

In our study, the combination of vincristine and HAART was generally well tolerated, although peripheral neuropathy was a dose-limiting factor in some patients. We found a baseline peripheral neuropathy rate of 50%. The prevalence of HIV-related peripheral neuropathy varies in different published series from 9–63%. References [19–24], with higher rates seen in those with more advanced immunosuppression and those on HAART. A similarly high incidence of neuropathy was reported in a large cross-sectional Ethiopian study, with the majority of cases found in those receiving HAART [24]. Likely contributing factors in our series were stavudine-containing HAART, prior tuberculosis treatment, and HIV neuropathy. Peripheral neuropathy is a particular concern in this patient population, causing significant morbidity with a limited choice of agents available for symptom management.

16 patients died during the six-month study period, including 6 early deaths that occurred within 6 weeks of recruitment. The median PFS in the ITT analysis was 30 weeks in our study, which is comparable with survival data reported by Olweny et al. [17]. Additional analysis presuming all defaulted patients who had died revealed a median progression-free survival of 24.5 weeks. This emphasizes the need for taking a holistic approach to patient management, aiming to improve quality of life for those in whom life expectancy is limited. Cause of death was difficult to ascertain in most cases given the majority of patients died at home. There was a trend towards a lower baseline CD4 count in those who died during the study. Given no clear association between lower baseline CD4 counts and KS progression, it is possible that opportunistic infections may have contributed to deaths more than progressive KS during the study period. Immune reconstitution inflammatory syndrome (IRIS) is well described in KS [25] and is another potential cause for deterioration in patients with KS receiving HAART. It is of interest that the baseline CD4 count of 263 in our study is somewhat higher than previously published. In similar cohorts [17, 26], further study regarding the causes of death in this group would be of interest.

In this study we rigorously attempted to contact patients who failed to attend appointments firstly by telephone and then by home visits. It is common cultural practice in Malawi to travel home to the family village as people near the end of their life. This may have contributed to at least 3 patients being lost to follow up. 3 others were known to have potentially life threatening conditions at the point of last contact—one with a large cerebrovascular accident, one with Stevens-Johnsons-syndrome thought secondary to HAART, and one with proven gastrointestinal KS. A prior study of patients on an antiretroviral programme in Lilongwe, Malawi, found that of those lost to follow-up, 41% were later found to have died [27]. It is likely that death was the cause of several defaults in this study.

Due to lack of access to prompt histopathological services, the diagnosis of KS in this study was made clinically by two experienced physicians. Histological confirmation would have been ideal if feasible.

The APCA POS tool has been validated for use in an African setting. We utilized it in this survey in an attempt to measure palliative care outcomes. Whilst some trends could be elucidated, we observed that the patients and families in our study found the questionnaire difficult to complete. They struggled to use numerical rating scales to express the magnitude of a subjective experience, even with the assistance of a research nurse. More refinement in such assessment tools is needed before they can be used reliably in our population group.

## 6. Conclusion

HIV-related KS is a common problem in Malawi. The combination of vincristine and ART is a feasible option for the treatment of patients with moderate-advanced disease in a low resource setting and is associated with good response rates; however, peripheral neuropathy is a dose-limiting factor. Given the poor prognosis in this patient group, adequate palliative care remains of upmost importance. Optimal utilization of a palliative care assessment tool remains a challenge in our setting.

## Figures and Tables

**Table 1:** Baseline patient characteristics.

Characteristic	Baseline
Age, (median (IQR)), years	33.5 (28–41)
Male (%)	40 (80.0)
KPS, median (IQR)	70 (50–80)
CD4 count, median (IQR)	263 (119–408)
Peripheral neuropathy (%) Grade 1 (%) Sensory (%)	25 (50) 18 (72%) 21 (84%)
HAART at baseline (%) Median time on HAART prior to recruitment (range)	37 (74%) 6.8 months (1–60)

## Appendix

### A. African Palliative Outcome Scale(APOS)

**Table:**

Study number _________________ Staff name_____________ Date ________________ POS no 1st□ 2nd□ 3rd□ 4th□ 5th□ 6th□	
Ask the patient	
Q1. Please rate your pain (from 0 = no pain to 5 worst/overwhelming pain) during the last 3 days	
Q2. Have any other symptoms (e.g., nausea, coughing or constipation) been affecting how you feel in the last 3 days?	
Q3. Have you been feeling worried about your illness the past 3 days?	
Q4. Over the past 3 days, have you been able to share how you are feeling with your family or friends?	
Q5. Over the past 3 days have you felt that life was worthwhile?	
Q6. Over the past 3 days, have you felt at peace?	
Q7. Have you had enough help and advice for your family to plan for the future?	
Ask the family carer	
Q8. How much information have you and your family been given?	
Q9. How confident does the family feel caring for ____?	
Q10. Has the family been feeling worried about the patient over the last 3 days?	
Possible responses	
A1. 0 (no pain)–5 (worst/overwhelming pain) 0 □ 1 □ 2 □ 3 □ 4 □ 5 □	
A2. 0 (not at all)–5 (overwhelmingly) 0 □ 1 □ 2 □ 3 □ 4 □ 5 □	
A3. 0 (not at all)–5 (overwhelming worry) 0 □ 1 □ 2 □ 3 □ 4 □ 5 □	
A4. 0 (not at all)–5 (yes, I've talked freely) 0 □ 1 □ 2 □ 3 □ 4 □ 5 □	
A5. 0 (no, not at all)–5 (yes, all the time) 0 □ 1 □ 2 □ 3 □ 4 □ 5 □	
A6. 0 (no, not at all)–5 (Yes, all the time) 0 □ 1 □ 2 □ 3 □ 4 □ 5 □	
A7. 0 (not at all)–5 (as much as wanted) 0 □ 1 □ 2 □ 3 □ 4 □ 5 □	
A8. 0 (none)–5 (as much as wanted) N/A □ 0 □ 1 □ 2 □ 3 □ 4 □ 5 □	
A9. 0 (not at all)–5 (very confident) N/A □ 0 □ 1 □ 2 □ 3 □ 4 □ 5 □	
A10. 0 (not at all)–5 (severe worry) N/A □ 0 □ 1 □ 2 □ 3 □ 4 □ 5 □	
